# Localizing spontaneously hemostatic colonic diverticular bleeding using VISCOCLEAR gel: A case report

**DOI:** 10.1016/j.amsu.2021.103141

**Published:** 2021-12-04

**Authors:** Daisuke Suto, Masashi Yoshida, Takaaki Otake, Eiichiro Ichiishi, Kiichi Sato, Yosuke Osawa, Hirotoshi Ebinuma, Hironori Odaira, Yutaka Suzuki, Yutaka Kohgo

**Affiliations:** a Department of Gastroenterology, International University of Health and Welfare Hospital, 537-3 Iguchi, Nasushiobara, Tochigi, 329-2763, Japan; b Department of Surgery, International University of Health and Welfare Hospital, 537-3, Iguchi, Nasushiobara, Tochigi, 329-2763, Japan; c Department of Gastroenterology and Hepatology, International University of Health and Welfare, Narita Hospital, 852, Hatagata, Narita, Chiba, 286-8520, Japan

**Keywords:** Colonic diverticular bleeding, VISCOCLEAR, Colonoscopy, Hemostatic clipping, Gastrointestinal bleeding

## Abstract

Colonic diverticular bleeding is the most common type of gastrointestinal bleeding. We report a case of an 82-year-old man with a chief complaint of melena. Enhanced computed tomography showed multiple diverticula, and water-assisted colonoscopy could not help identify the diverticulum responsible for bleeding. We injected VISCOCLEAR, a novel gel formulation, into the digestive tract endoscopically and successfully localized the bleeding point. Moreover, the use of VISCOCLEAR secured a clear visual field with reduced glare, as seen in the digital endoscopic image. Subsequently, we performed hemostatic clipping. The course after the endoscopic treatment was unremarkable. In this case, we could identify the exposed bleeding vessels in the diverticulum using VISCOCLEAR and perform hemostatic clipping. We intend to evaluate the effectiveness of VISCOCLEAR further by analyzing a series of cases.

## Highlights

• An 80-year-old man presented with melena and colonic diverticular bleeding.
• VISCOCLEAR novel gel formulation helped successfully localize the bleeding point.
• VISCOCLEAR helped secure a clear visual field with reduced glare.
• The course after the endoscopic treatment was unremarkable.

## 1 Introduction

VISCOCLEAR (Otsuka Pharmaceutical Factory, Inc., Tokushima, Japan) is a novel homogenous gel formulation used in patients with colonic diverticular bleeding to secure a clear visual field during endoscopy. The gel consists of gelling agents, concentrated glycerin, and purified water without electrolytes. Yano et al. [1] first reported gel immersion endoscopy in 2016. They demonstrated that the viscosity of the gel prevents the mixing of the gel and blood, thereby helping us visualize the bleeding point. The efficacy of the gel immersion method for endoscopic hemostasis has been previously reported [2,3]. Although VISCOCLEAR is generally used to immerse the colonic lumen, some clinicians have also found it helpful in identifying duodenal ulcer bleeding [4]. However, it is only used for cleaning and immersing the diverticular lumen. Identifying the bleeding diverticulum among other diverticula remains challenging, even using colonoscopy. Jensen et al. [5] reported that diverticular bleeding was detected by colonoscopy in 21% of cases. Here, we report a case of spontaneous hemostatic diverticular bleeding identified endoscopically with the help of VISCOCLEAR. The case has been reported in line with the SCARE checklist [6].

## 2 Presentation of case

**Fig. 1:**
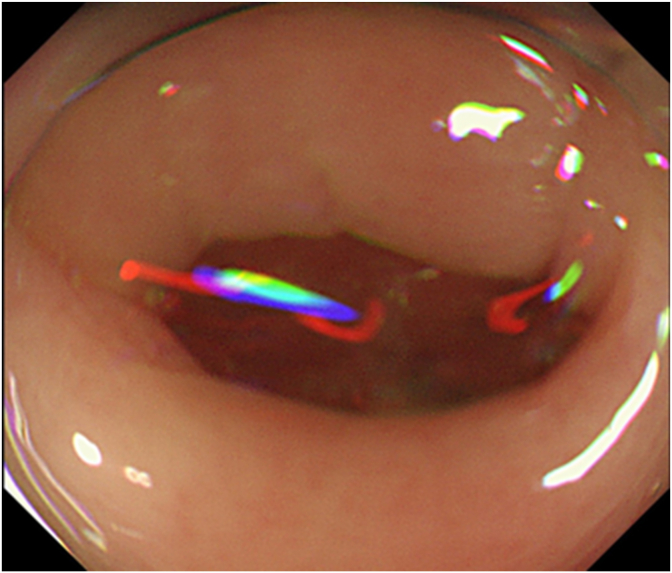
Endoscopy was performed after spontaneous hemostasis. An endoscopic image of the ascending colon washed with water does not show the diverticulum responsible for the bleeding.

**Fig. 2:**
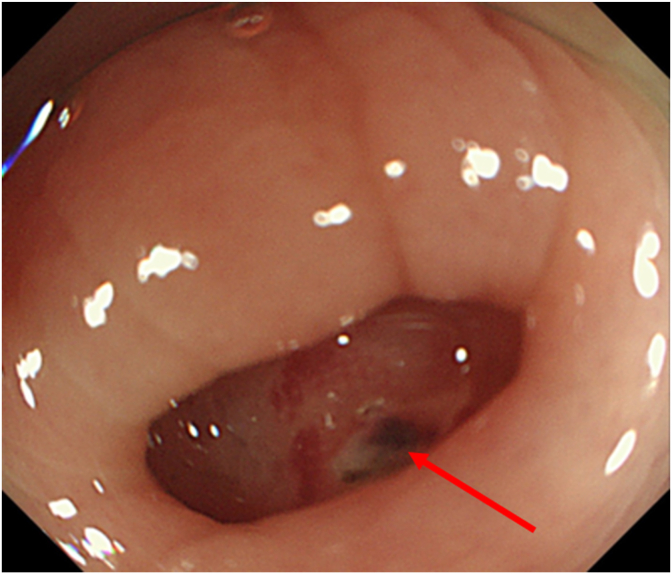
An endoscopic image of ascending colon with VISCOCLEAR applied, showing a clear visual field with the identified exposed vessels (red arrow). (For interpretation of the references to colour in this figure legend, the reader is referred to the Web version of this article.)

**Fig. 3:**
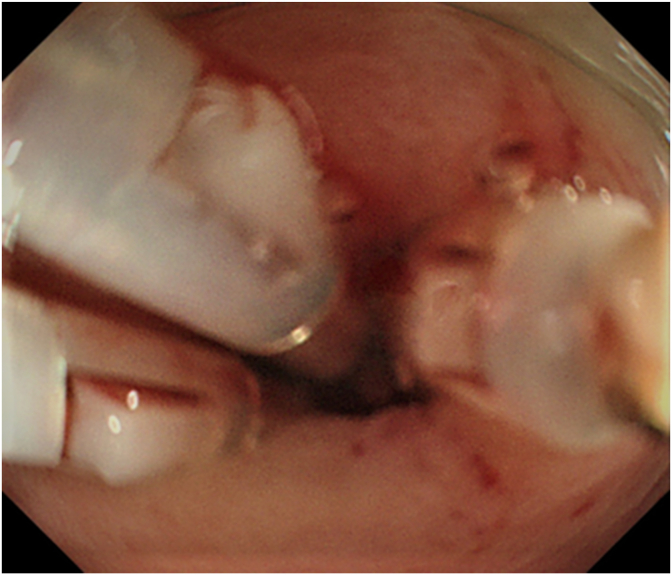
Endoscopic hemostatic clipping of diverticulum after washing with VISCOCLEAR.

An 82-year-old man presented to the Digestive Disease Center of the International University of Health and Welfare Hospital with the chief complaint of melena. An enhanced computed tomography scan of the abdomen revealed multiple diverticula in the ascending colon. However, the source of the bleeding could not be identified. Three days after admission, the patient was prepped with polyethylene glycol-electrolyte solution and underwent colonoscopy (PCF–H290I, Olympus Tokyo, Japan). A soft hood (D201‐12704, Olympus Medical Systems) was attached to the endoscope. Multiple diverticula were found in the ascending colon; however, no blood accumulation was detected. Despite washing with water, the diverticular lesion responsible for bleeding remained unidentified (Fig. 1). Therefore, we injected VISCOCLEAR through the endoscope (Fig. 2). We could locate the vessel responsible for bleeding after covering the diverticulum with VISCOCLEAR. Subsequently, hemostatic clipping was performed (Fig. 3) using hemoclips (HX-600-090L Olympus Optical Co. Ltd., Tokyo, Japan). The postoperative course following the endoscopic treatment was uneventful.

## 3 Discussion

Colonic diverticular bleeding is the most common type of gastrointestinal bleeding [7]. The risk of colonic diverticular bleeding increases with colonic diverticulosis [8]. When the bleeding stops spontaneously during a colonoscopy, localization of the bleeding point becomes difficult because the exposed bleeding vessel disappears within 24 hours in about one-third of cases [9]. Moreover, the exposed bleeding vessel in the diverticulum is tiny, and it is difficult to observe the entire interior of the diverticulum. Using a translucent hood at the tip and a jet cleaning system is suggested to improve the observation conditions. Previous reports suggest a detection rate between 15% and 85% for bleeding sources [10,11]. Here, we report a case of spontaneous hemostatic diverticular bleeding in the colon successfully and endoscopically identified using VISCOCLEAR but not with water-assisted endoscopy. Covering the diverticular lumen of the colon is easier with VISCOCLEAR because the diverticular lumen of the colon is smaller than that of the stomach and duodenum. In addition to a clear visual field, the gel reduced the reflection of light.

As a key limitation of this report, this experience is from a single center, and a difference in technique might lead to different results elsewhere. However, it was apparent from our observations that Viscoclear might help identify bleeding points for spontaneously hemostatic diverticular bleeding.

## 4 Conclusion

In this case, we could identify the exposed bleeding vessels in the diverticulum using VISCOCLEAR and perform hemostatic clipping. We intend to evaluate the effectiveness of VISCOCLEAR further by analyzing a series of cases.

## Provenance and peer review

Not commissioned, externally peer-reviewed

## Annals of medicine and surgery

The following information is required for submission. Please note that failure to respond to these questions/statements will mean your submission will be returned. If you have nothing to declare in any of these categories then this should be stated.

### Ethical approval

The study was approved by the Ethics Committee of the International University of Health and Welfare Hospital [approval number: 21-B-16].

### Please state any sources of funding for your research

None.

### Author contribution

All authors contributed equally to the manuscript.

### Consent

Written informed consent was obtained from the patient to publish this case report and any accompanying images.

### Registration of research studies

Not applicable as this is not a clinical trial. It is a case report.

### Guarantor

Daisuke Suto, First Author.

Masashi Yoshida, Senior Author.

## Declaration of competing interest

None.
